# Dynamic Changes in Endothelial Glycocalyx and Inflammatory Response in Patients with Acute Ischemic Stroke Treated with Mechanical Thrombectomy: Pathophysiological Aspects and Clinical Implications

**DOI:** 10.3390/neurolint18050077

**Published:** 2026-04-23

**Authors:** Berya Günay, Samyuktha Ramesh Dhayanand, Marijana Matas, Vlatka Sotosek, Lara Baticic

**Affiliations:** 1 Faculty of Medicine, University of Rijeka, 51000 Rijeka, Croatia; berya.gunay@uniri.hr (B.G.); sramesh@uniri.hr (S.R.D.); 2 Department of Anesthesiology, Reanimatology and Intensive Care Medicine, University Hospital Center Zagreb, 10000 Zagreb, Croatia; marijana.matas@uniri.hr; 3 Department of Anesthesiology, Reanimatology, Emergency and Intensive Care Medicine, Faculty of Medicine, University of Rijeka, 51000 Rijeka, Croatia; 4 Department of Clinical Medical Sciences I, Faculty of Health Studies, University of Rijeka, 51000 Rijeka, Croatia; 5 Department of Medical Chemistry, Biochemistry and Clinical Chemistry, Faculty of Medicine, University of Rijeka, 51000 Rijeka, Croatia

**Keywords:** acute ischemic stroke, endothelial glycocalyx, endothelium, inflammation, mechanical thrombectomy

## Abstract

Acute ischemic stroke (AIS) is characterized by complex interactions among vascular occlusion, endothelial injury, and inflammatory activation, which collectively influence clinical outcomes. Increasing attention has focused on the endothelial glycocalyx, a critical regulator of vascular permeability, mechanotransduction, and inflammatory signaling. Disruption of the endothelial glycocalyx during ischemia and subsequent reperfusion contributes to blood–brain barrier (BBB) dysfunction and secondary brain injury. Mechanical thrombectomy has emerged as the reference standard treatment for large vessel occlusion in AIS. This review synthesizes current evidence on endothelial glycocalyx degradation and associated inflammatory cascades in cute ischemic stroke, with particular emphasis on patients undergoing mechanical thrombectomy. We examine the mechanisms underlying endothelial and BBB injury, ischemia–reperfusion-mediated vascular dysfunction, and systemic inflammatory responses (SIRS). In addition, the potential clinical relevance of circulating biomarkers indicative of endothelial glycocalyx shedding and endothelial damage is discussed. By integrating molecular pathophysiology with contemporary reperfusion strategies, this review highlights the importance of endothelial protection as a potential adjunct to mechanical thrombectomy. While mechanical thrombectomy remains the gold standard therapy for AIS due to large vessel occlusion, targeting endothelial glycocalyx integrity and post-reperfusion inflammation may represent a promising approach to optimizing neurological outcomes and reducing complications. Further research is required to elucidate specific pathophysiological mechanisms and to develop targeted therapeutic strategies aimed at reducing stroke-related morbidity and mortality.

## 1. Introduction

Acute ischemic stroke (AIS) remains a leading cause of mortality and long-term disability worldwide, imposing a substantial burden on patients, caregivers, and healthcare systems. According to the Global Burden of Disease (GBD) Study, stroke ranked as the third leading cause of death and the fourth leading cause of disability globally in 2021 [1,2,3,4,5,6]. AIS accounts for approximately 85% of all cases, underscoring its major public health impact despite advances in prevention and acute management. The American Heart Association defines AIS as an episode of neurological dysfunction caused by focal cerebral, spinal, or retinal infarction within a defined vascular territory, with symptoms lasting more than 24 h or resulting in death. Diagnosis is based on clinical evaluation supported by neuroimaging evidence of focal ischemic injury, typically using computed tomography or magnetic resonance imaging [7,8,9].

In recent years, increasing attention has been directed toward the endothelial glycocalyx, a gel-like, carbohydrate-rich layer lining the luminal surface of endothelial cells. Composed primarily of proteoglycans, glycosaminoglycans, and associated plasma proteins, the endothelial glycocalyx plays a pivotal role in maintaining blood–brain barrier (BBB) integrity and vascular homeostasis. Within the central nervous system (CNS), it regulates vascular permeability, mechanotransduction, shear-stress sensing, leukocyte adhesion, and inflammatory signaling, thereby contributing to cerebral blood flow regulation and neurovascular coupling [10,11,12,13]. Disruption of the endothelial glycocalyx during cerebral ischemia and subsequent reperfusion is characterized by enzymatic degradation and shedding of its structural components, leading to increased endothelial permeability, activation of inflammatory cascades, and heightened susceptibility to hemorrhagic complications. Accordingly, glycocalyx degradation is increasingly recognized as a key mediator of vascular dysfunction in AIS and other neurological disorders [14,15,16,17].

AIS is now widely understood as a disorder of the neurovascular unit, in which endothelial dysfunction and inflammation play central roles. The neurovascular unit consists of neurons, astrocytes, and brain microvascular endothelial cells that function in a highly coordinated manner. Cerebral ischemia triggers both local and systemic inflammatory responses, characterized by activation of resident microglia and astrocytes, recruitment of peripheral immune cells, and release of pro-inflammatory cytokines, chemokines, and reactive oxygen species (ROS) [18,19,20,21,22,23]. These processes contribute to BBB breakdown, cerebral edema, and secondary neuronal injury, further impairing cerebral perfusion through endothelial activation and microvascular dysfunction [24,25]. Persistent endothelial injury exacerbates microcirculatory failure and amplifies tissue damage beyond the initial ischemic core.

For AIS caused by large vessel occlusion, mechanical thrombectomy has become the standard of care, enabling rapid and effective recanalization and significantly improving functional outcomes and survival compared with intravenous thrombolysis alone [18,19,20,21,22,23,24,25]. However, the abrupt restoration of blood flow may precipitate ischemia–reperfusion injury (IRI), characterized by oxidative stress, endothelial activation, and intensified inflammatory responses [26,27,28,29,30,31]. These processes can contribute to complications such as intracerebral hemorrhage, systemic inflammatory response syndrome (SIRS), and multiple organ dysfunction syndrome (MODS), thereby limiting the full benefit of successful recanalization [24,25].

In this context, the endothelial glycocalyx represents a critical interface linking vascular dysfunction, inflammation, and reperfusion-related injury. Glycocalyx degradation enhances leukocyte–endothelium interactions, exposes adhesion molecules, and contributes to BBB disruption, edema formation, and thromboinflammatory responses, particularly following rapid recanalization [10,11,12,13,14,15,16,17,28,29,30,31]. Circulating biomarkers of endothelial glycocalyx shedding provide valuable insight into the severity of endothelial injury, disease prognosis, and therapeutic response [32,33,34,35,36,37].

The aim of this narrative review is to provide a comprehensive overview of the structural and functional role of the endothelial glycocalyx in AIS, with particular emphasis on its dynamic alterations during ischemia and reperfusion, its contribution to endothelial dysfunction and inflammatory responses, and its potential as a target for prognostic assessment and therapeutic intervention in patients undergoing mechanical thrombectomy remains a leading cause of mortality and long-term disability worldwide, placing a substantial burden on patients, caregivers, and healthcare systems. According to the Global Burden of Disease (GBD) Study, stroke was the third leading cause of death and the fourth leading cause of disability globally in 2021 [1,2,3,4,5,6]. Ischemic stroke accounts for approximately 85% of all stroke cases and represents a major public health challenge despite advances in prevention and acute management. The American Heart Association defines ischemic stroke as an episode of neurological dysfunction caused by focal cerebral, spinal, or retinal infarction within a defined vascular territory, with symptoms persisting for more than 24 h or resulting in death. Diagnosis relies on clinical assessment supported by neuroimaging evidence of focal ischemic injury, typically using computed tomography or magnetic resonance imaging [7,8,9]. In recent years, increasing attention has been directed toward the endothelial glycocalyx, a gel-like, carbohydrate-rich layer that coats the luminal surface of endothelial cells. Composed primarily of proteoglycans, glycosaminoglycans, and associated plasma proteins, the endothelial glycocalyx plays a pivotal role in maintaining blood–brain barrier (BBB) integrity and vascular homeostasis. Within the central nervous system (CNS), the endothelial glycocalyx regulates vascular permeability, mechanotransduction, shear-stress sensing, leukocyte adhesion, and inflammatory signaling, thereby contributing to cerebral blood flow regulation and neurovascular coupling [10,11,12,13]. Disruption of the endothelial glycocalyx during cerebral ischemia and subsequent reperfusion is characterized by enzymatic degradation and shedding of its structural components, leading to increased endothelial permeability, activation of inflammatory cascades, and heightened susceptibility to hemorrhagic complications. Endothelial glycocalyx degradation has been implicated in a range of pathological conditions, including AIS and traumatic brain injury, and is increasingly recognized as a key mediator of vascular dysfunction [14,15,16,17]. Cerebral ischemia initiates both local and systemic inflammatory responses, characterized by the activation of resident microglia and astrocytes, recruitment of peripheral immune cells, and the release of pro-inflammatory cytokines, chemokines, and reactive oxygen species (ROS) [18,19,20,21,22,23]. These processes contribute to BBB breakdown, cerebral edema, and secondary neuronal injury, further impairing cerebral perfusion through endothelial activation and microvascular dysfunction [24,25]. Persistent endothelial injury exacerbates microcirculatory failure and amplifies tissue damage beyond the initial ischemic core. In AIS, endothelial glycocalyx integrity establishes a key connection linking perfusion failure to endothelial activation, BBB disruption, ischemia–reperfusion injury and thromboinflammation. Reperfusion and ischemia elicit oxidative stress and release inflammatory mediators which enzymatically degrades the endothelial glycocalyx. This enhances leukocyte–endothelium interactions while exhibiting endothelial adhesion particles. Resulting in BBB dysfunction, hemorrhagic risk, edema and inflammatory cascades, especially in rapid recanalization with mechanical thrombectomy [10,11,12,13,14,15,16,17,28,29,30,31].

## 2. Pathophysiological Background

In AIS, endothelial glycocalyx integrity establishes a key connection linking perfusion failure to endothelial activation, BBB disruption, IRI and thromboinflammation. Reperfusion and ischemia elicit oxidative stress and release inflammatory mediators which enzymatically degrades the endothelial glycocalyx. This enhances leukocyte–endothelium interactions while exhibiting endothelial adhesion particles. Resulting in BBB dysfunction, hemorrhagic risk, edema and inflammatory cascades, especially in rapid recanalization with mechanical thrombectomy [10,11,12,13,14,15,16,17,28,29,30,31].

### 2.1. Endothelial Glycocalyx: Structure, Function, and Integrity in Cerebral Circulation

The endothelial glycocalyx is a gel-like, highly dynamic network of polysaccharides that extends from the endothelial cell membrane into the vascular lumen and represents the luminal component of the neurovascular barrier, serving as a protective and regulatory interface between blood flow and the vascular wall [10,11,12,13]. As a specialized form of extracellular matrix, it is characterized by a delicate balance between synthesis and degradation, which determines its composition, thickness, and functional integrity. Circulating plasma proteins further contribute to its structural stability. All blood vessels are lined by endothelial cells whose luminal surfaces are coated with a fibrillar meshwork that constitutes the endothelial glycocalyx. Its major components include proteoglycans (core proteins covalently linked to glycosaminoglycans, GAGs), glycoproteins (such as integrins and selectins), and glycolipids (glycosphingolipids) [38,39,40,41,42,43,44]. The proteoglycan side chains consist of five primary types of GAGs: hyaluronic acid, keratan sulfate, heparan sulfate, dermatan sulfate, and chondroitin sulfate. Heparan sulfate is particularly important for multiple biological processes, including growth factor binding, regulation of cell proliferation and migration, immune cell adhesion to the endothelium, and modulation of inflammatory responses via cytokine interactions. The two principal classes of proteoglycans in the glycocalyx are syndecans and glypicans; syndecans are transmembrane proteins involved in intracellular signaling, whereas glypicans are anchored to lipid rafts and contribute to structural organization and functional regulation [38,39,40,41,42,43,44].

Functionally, the endothelial glycocalyx plays a central role in modulating cell signaling, sensing the extracellular environment, and maintaining vascular permeability. It acts as a mechanosensor by detecting shear stress from blood flow and converting mechanical stimuli into intracellular signaling cascades that regulate gene expression, including that of transporters and tight junction proteins [45,46,47,48,49]. Through its interaction with shear stress, the endothelial glycocalyx contributes to nitric oxide (NO) production, a key regulator of vascular tone [50,51,52,53,54,55]. Its negatively charged structure confers charge selectivity, limiting the passage of negatively charged molecules across the endothelium and contributing to barrier function. Furthermore, circulating molecules can bind to endothelial glycocalyx components, enhancing its stability and regulating local concentrations at the endothelial surface, thereby facilitating intracellular signal transduction and suppressing inflammatory and coagulation pathways. The endothelial glycocalyx also serves as a structural barrier that shields adhesion molecules such as intracellular adhesion molecule (ICAM), vascular cell adhesion molecule (VCAM), and platelet endothelial cell adhesion molecule (PECAM), preventing inappropriate leukocyte and platelet adhesion under physiological conditions. Through coordinated interactions with enzymatic and signaling molecules, including hyaluronan synthase 2 (HAS2), CD44, and Src kinase, it regulates cytoskeletal dynamics, endothelial permeability, and inflammatory signaling. By maintaining an anti-inflammatory endothelial phenotype and modulating immune cell interactions, the glycocalyx acts as the first interface between circulating blood components and the vascular wall.

Within the CNS, the endothelial glycocalyx is an essential component of the neurovascular unit and plays a critical role in maintaining the integrity and function of the BBB. The BBB is a dynamic and highly specialized barrier composed of brain microvascular endothelial cells, tight junctions, basement membrane, pericytes, and astrocytic end-feet, which together regulate solute exchange, provide metabolic support, and protect neural tissue from circulating toxins, pathogens, and peripheral immune cells [24,25,45,46,47,48,49]. The endothelial glycocalyx contributes to BBB function by regulating molecular trafficking on the luminal endothelial surface, maintaining vascular permeability, and controlling interactions between circulating cells and the endothelium. It also participates in the regulation of cerebral blood flow and inflammatory responses, thereby supporting CNS homeostasis under both physiological and pathological conditions [10,11,12,13].

Disruption of the endothelial glycocalyx is closely associated with BBB dysfunction. endothelial glycocalyx degradation leads to increased vascular permeability and loss of charge selectivity, facilitating the infiltration of inflammatory cells and circulating factors into brain tissue. This process is accompanied by tight junction disorganization, basement membrane remodeling, pericyte detachment, and astrocytic end-foot dysfunction, resulting in a structurally and functionally impaired BBB [24,25,45,46,47,48,49]. Such alterations are characteristic of AIS and other neurological disorders, where endothelial glycocalyx breakdown contributes to neuroinflammation, edema formation, and secondary brain injury.

**Figure 1:**
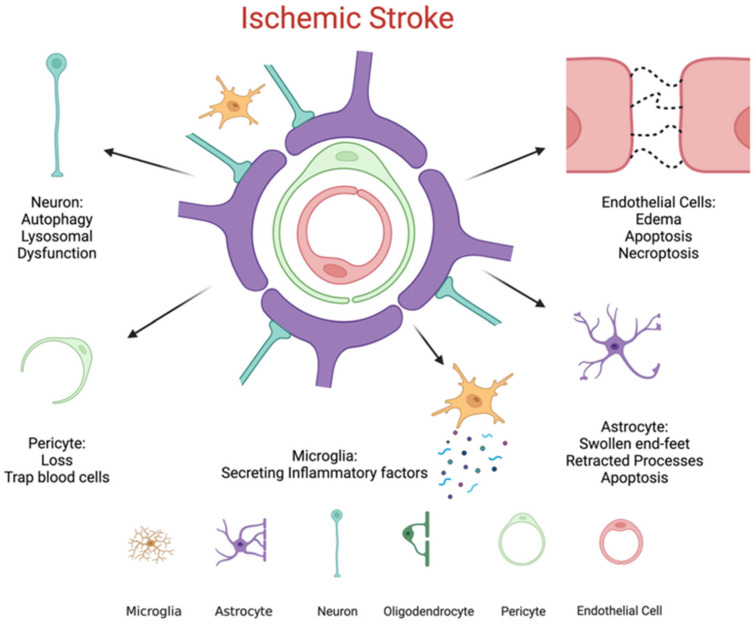
Cellular components of the blood–brain barrier and ischemia-induced alterations. The blood–brain barrier (BBB) is composed of endothelial cells, pericytes, astrocytic end-feet, and extracellular matrix components that maintain selective permeability. During ischemic stroke, these cellular constituents and associated signaling molecules are disrupted, leading to endothelial dysfunction, degradation of tight junction proteins, and breakdown of the endothelial membrane, ultimately compromising BBB integrity [45].

Overall, the endothelial glycocalyx plays a fundamental role in preserving vascular integrity by acting as a physical and biochemical barrier, a mechanotransducer of shear forces, and a regulator of endothelial permeability and inflammation. Its integrity is crucial for maintaining BBB function and overall cerebral vascular homeostasis [46,47,48,49,50,51,52,53,54,55]. Cellular components of the BBB and ischemia-induced alterations. Modifications in endothelial cells and endothelial glycocalyx structure following ischemic insult are illustrated in Figure 1. 

### 2.2. Mechanisms of Endothelial Glycocalyx Degradation During Ischemia

When the endothelial glycocalyx breaks down and sheds, it loses its ability to interact with circulating proteins, cells, and other blood components. This disruption promotes endothelial dysfunction, increases vascular inflammation, and enhances the risk of thrombosis. Normally, molecules in circulation can bind to the endothelial glycocalyx, reinforcing its structural integrity, preventing degradation, and modulating the expression of specific cell surface receptors. Through these interactions, the endothelial glycocalyx facilitates intracellular signaling and helps maintain anti-inflammatory and anticoagulant states within the vasculature [50,51,52,53,54,55].

An implication of endothelial glycocalyx degradation has been made in a variety of pathological conditions, including AIS and traumatic brain injury. The endothelial glycocalyx in AIS is mainly characterized by enzymatic breakdown caused by IRI, especially by activation of matrix metalloproteinases. By disrupting tight junction protein expression and endothelial integrity, endothelial glycocalyx loss affects mechanotransduction in hypo perfused situations and plays a major role in BBB disruption. Structural shedding and quantifiable layer thinning are indicators of endothelial glycocalyx damage in traumatic brain injury. While decreases in endothelial glycocalyx thickness and density, even following recurrent mild injury, are linked to later behavioral abnormalities, elevated circulating syndecan-1 indicates systemic release of endothelial glycocalyx components. While AIS is mostly enzyme-mediated and traumatic brain injury exhibits significant physical shedding and structural deterioration, both disorders collectively emphasize endothelial glycocalyx disruption as a key characteristic of neurovascular injury. These findings suggest that the endothelial glycocalyx is not merely a passive barrier but an active participant in disease progression, contrasting with its protective role under physiological conditions. For instance, studies have observed endothelial glycocalyx layer degradation in patients with cerebral small vessel disease who exhibit white matter lesions. Similarly, endothelial glycocalyx impairment is closely associated with IRI, as seen in AIS [14,15,16,17].

A key pathological mechanism in small vessel disease is cerebral endothelial cell dysfunction. Endothelial cell failure compromises the integrity of the BBB and disrupts cell–cell communication within the neuro-glial-vascular unit, ultimately damaging surrounding brain tissue. This dysfunction can arise from intrinsic factors, such as genetic susceptibility, and extrinsic factors, including diabetes, hypertension, and smoking [56,57,58,59,60,61].

**Figure 2:**
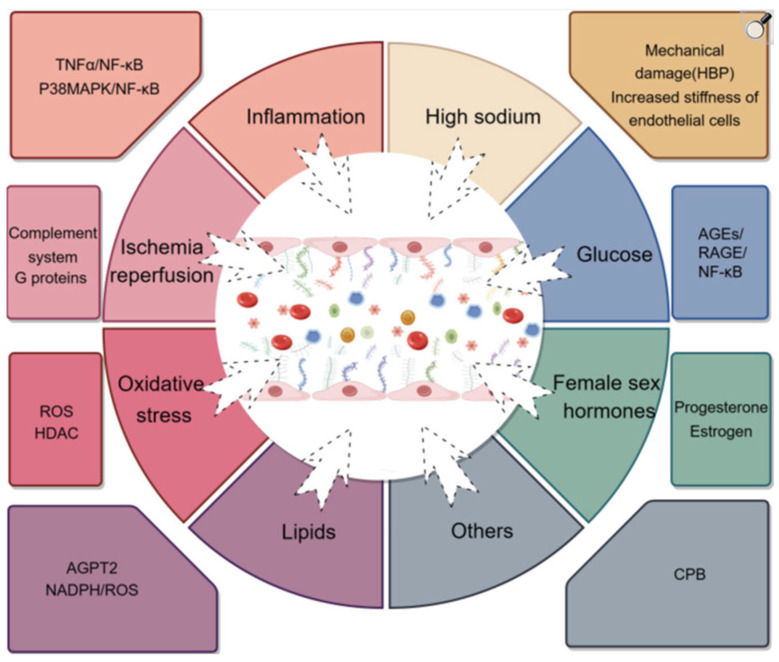
Factors influencing endothelial glycocalyx degradation. The endothelial glycocalyx is a dynamic layer critical for vascular barrier function, mechanotransduction, and anti-inflammatory signaling. Its integrity can be disrupted by multiple variables, including ischemia–reperfusion injury (IRI), oxidative stress, inflammatory mediators, hyperglycemia, and enzymatic degradation, leading to endothelial dysfunction and increased vascular permeability. TNFα—tumor necrosis factor alpha; NFκB—nuclear factor kappa B.

The primary drivers of endothelial glycocalyx loss are excessive enzymatic shedding and impaired synthesis. Enzymes that degrade the endothelial glycocalyx, such as MMPs, heparanase, a disintegrin and metalloproteinase family members, and hyaluronidases, are frequently overexpressed under pathological conditions. Concurrently, conditions that suppress endothelial glycocalyx biosynthesis further contribute to its reduction, leading to decreased endothelial glycocalyx expression and compromised vascular homeostasis (Figure 2) [50,51,52,53,54,55].

The endothelial glycocalyx experiences a two-stage process of partial regeneration and degradation during ischemic brain injury. First, ischemia and reperfusion cause important endothelial glycocalyx components like syndecan-1 and hyaluronan to shed quickly, which causes noticeable thinning and structural disruption. The repaired layer frequently resembles a sparser, peripheral-type structure rather than the dense, specialized BBB-specific mesh, even though the endothelial glycocalyx can anatomically regain thickness throughout the recovery phase. This breakdown is reflected in circulating fragments like syndecan-1, which could be used as biomarkers of the extent of harm. Functionally, significant BBB dysfunction results from endothelial glycocalyx degradation. When its physical and electrostatic barrier qualities are lost, permeability rises, edema is encouraged, and neurotoxic chemicals can enter. Additionally, it is linked to oxidative stress, leukocyte recruitment, pro-inflammatory endothelium activation with adhesion molecule exposure, increased caveolae-mediated transcytosis, and compromised mechanotransduction. The endothelial glycocalyx’s regulatory and protective specialization is still changed, even though structural thickness may partially restore. This could put the brain at risk for chronic inflammation and secondary damage [10].

The role of proteases and inflammatory signaling pathways induced by ischemia–reperfusion (e.g., NF-κB linked transcriptional programs) using enzymes such as MMPs and heparinase, is to upregulate endothelial glycocalyx shedding. Endothelial glycocalyx degradation leads to vascular injury in addition to actively accelerate inflammatory cell recruitment and BBB permeability alteration by facilitating access to adhesion receptors and declining shear-stress-mediated protection [28,29,30,31,50,51,52,53,54,55,62].

### 2.3. Circulating Biomarkers Reflecting Endothelial Glycocalyx Shedding

Vascular homeostasis is maintained by the endothelial glycocalyx, a layer of carbohydrates that lines the vascular endothelium that controls permeability, inflammatory transduction, and leukocyte adherence. In several disorders, its breakdown has been linked to endothelial impairment and organ injury. Furthermore, hepran sulphate and syndecan-1 are two examples of endothelial glycocalyx-derived biomarkers which can be found in urine and blood and may provide insight into vascular damage. Importantly, these biomarkers have demonstrated diagnostic and prognostic value in various clinical settings, including infectious diseases (sepsis), acute respiratory distress syndrome, kidney diseases, cardiovascular disorders, trauma, supporting their role in therapeutic response monitoring. Additionally, they facilitate better risk classification, earlier detection, and individualized therapy due to their significant affiliation with illness severity and prognosis. This underlines the importance for enabling more accurate clinical judgements for a variety of medical ailments [32].

Numerous conditions like sepsis, pancreatitis, hypertension diseases induced by pregnancy, share endothelial glycocalyx damage as a common pathological feature along with syndecan-1 shedding. Three convergent downstream processes are started by this fundamental event: inflammation, immune thrombosis/coagulopathy, and enhanced permeability. Acute kidney injury and acute respiratory distress syndrome are two examples of how the processes work together to cause organ failure. Syndecan-1 in circulation is a detectable indicator of endothelial pathological alteration [32].

However, currently available analytical methods cannot definitively determine whether circulating endothelial glycocalyx fragments originate from the endothelium. As a result, syndecan-1 should be regarded as a marker of vascular injury without assuming it originates solely from endothelial cells. Moreover, vendor variability between inter-assay platforms, differences between serum and plasma, pre-analytical variables such as sample timing and processing conditions may affect measured concentrations. Consequently, reported reference values should be interpreted cautiously, as they may vary depending on patient population. Therefore, further investigation is needed to clarify the role of syndecan1 in clinical applications. Established standardized cutoff thresholds, and confirming its evaluation of effectiveness through randomized controlled trials are essential steps towards clinical implementation [32].

One phospholipid metabolite found in the membranes, spingosine-1-phosphate, can prevent endothelial glycocalyx from shedding and promote the formation of endothelial glycocalyx components. The phosphoinositide 3-kinase (PI3K) pathway is mediating the mechanism of S1P which stimulates the expression of syndecan-1 and incorporates chondroitin sulphate chains and heparan sulphate on endothelial cells [14,15,16,17].

### 2.4. Mechanical Thrombectomy

Mechanical thrombectomy enables rapid and effective recanalization of occluded cerebral vessels and has become a cornerstone therapy for AIS, particularly in cases caused by large vessel occlusion. Over the past decade, mechanical endovascular thrombectomy has fundamentally transformed the management of AIS, demonstrating substantial improvements in functional outcomes and survival [63]. Concurrent advancements in angiography systems and endovascular technologies have enhanced procedural safety and efficacy, while also improving patient and operator protection by reducing radiation exposure without compromising the effectiveness of thrombectomy procedures [64,65,66,67,68]. For many years, pharmacological thrombolysis with intravenous or intra-arterial administration of fibrinolytic agents, such as alteplase, represented the only evidence-based therapeutic approach for AIS. Although effective in selected patients, thrombolysis is limited by a narrow therapeutic window, contraindications, and modest recanalization rates in cases of large vessel occlusion. The introduction of mechanical endovascular recanalization has therefore marked a major paradigm shift in the management of AIS. Mechanical thrombectomy has become the standard of care for eligible patients, demonstrating significantly improved functional outcomes and reduced disability compared with pharmacological thrombolysis alone [3,4,5,6,7,8,9,10,11,12,13,14,15,16,17]. Large randomized controlled trials have established the efficacy of endovascular thrombectomy when performed within 6 h of stroke onset and, with appropriate patient selection using advanced imaging techniques, up to 24 h after symptom onset [69,70,71,72,73,74]. 

Mechanical thrombectomy achieves revascularization by mechanically removing obstructing thrombi from major intracranial arteries, particularly in large vessel occlusion, using minimally invasive endovascular techniques such as stent retrievers or direct aspiration catheters [75]. Successful reperfusion aims to restore cerebral blood flow partially or completely within the ischemic territory, thereby limiting infarct expansion, preserving the penumbral tissue, and promoting neurological recovery. Despite its clinical benefits, mechanical thrombectomy is also associated with IRI and vascular endothelial stress, underscoring the need for adjunctive strategies to optimize outcomes following successful recanalization [76]. Angiography plays a crucial role in the visualization of intracranial occlusions during mechanical thrombectomy, providing real-time imaging to guide endovascular interventions. Biplane angiography systems allow simultaneous imaging in two orthogonal planes, enabling precise localization of the target lesion and improved navigation of endovascular devices. This dual-plane capability is associated with enhanced procedural quality, reduced fluoroscopy time, and decreased use of contrast media, which collectively contribute to improved patient safety and procedural efficiency. In contrast, single-plane angiography systems, while more cost-effective and widely available, require separate acquisitions in each plane. This typically necessitates two distinct contrast injections and two X-ray exposures to obtain comparable anatomical information, potentially increasing procedure time, radiation dose, and contrast load [64,65,66,67,68]. The choice between single- and biplane systems therefore involves balancing resource availability, cost considerations, and the potential impact on procedural accuracy and safety. Prophylaxis, assessment, and intervention are crucial since ischemic stroke is the leading cause of dysfunction in adults and a significant illness for society. Since endovascular treatment has become the established reference method for treating large vessel occlusion, interventional specialists must be aware of the most significant side effects and how to handle them. Intracerebral and subarachnoid hemorrhages are examples of intracranial hemorrhages, which are serious consequences that greatly impair the clinical results. For this reason, early surveillance and effective management are pivotal. Considering there are few treatment options for cerebral air embolism, prevention is key [77,78,79,80,81,82].

**Figure 3:**
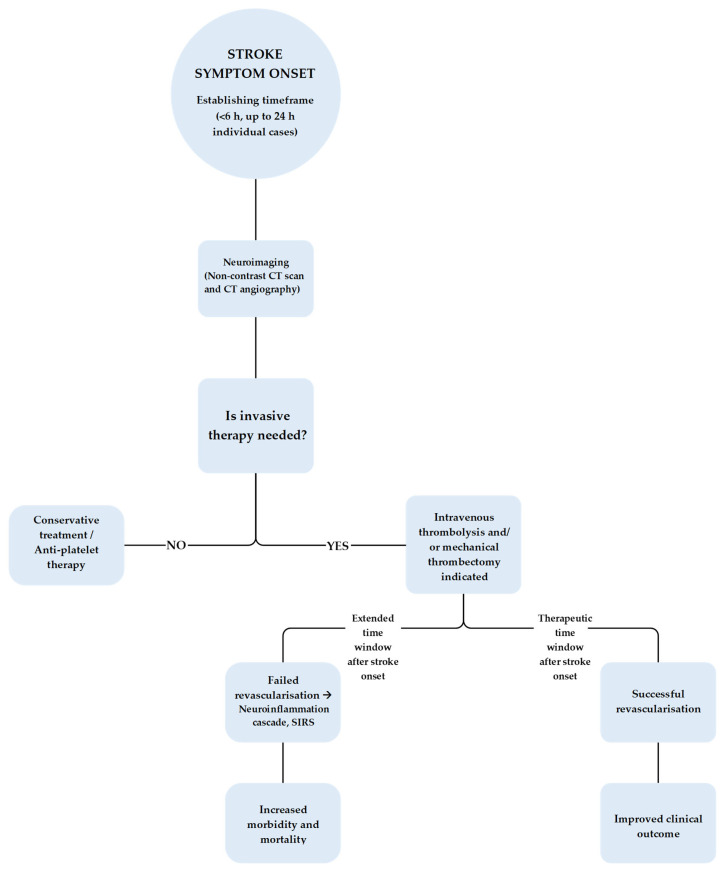
Algorithm for the acute management of patients with ischemic stroke and potential clinical outcomes.

Complications can worsen due to uncontrolled risk factors. Large vessel occlusions are in correlation with an increased rate of reoccurrence stroke due to intracranial atherosclerosis. Distal and medium sized vessel occlusions are also related to hypertension and hyperlipidemia [69,70,71,72,73,74]. Figure 3 illustrates the management algorithm for patients with AIS, therapeutic options such as thrombolysis and thrombectomy, and the potential clinical outcomes following each intervention.

### 2.5. Reperfusion Injury and Secondary Endothelial Damage

#### Complications and Therapeutic Implications

Clinically, IRI contributes to secondary brain injury following recanalization and may limit the benefits of reperfusion therapies. In the context of mechanical thrombectomy, endothelial glycocalyx disruption and endothelial dysfunction are increasingly recognized as key contributors to suboptimal post-procedural outcomes, including incomplete microvascular reperfusion, the no-reflow phenomenon, cerebral edema, and hemorrhagic transformation. Thus, preservation of endothelial glycocalyx integrity represents a critical determinant of successful tissue recovery despite macrovascular recanalization.

In severe cases, IRI can also trigger systemic inflammatory responses, including SIRS and MODS, thereby increasing overall morbidity and mortality. Current clinical strategies focus primarily on optimizing reperfusion, including minimizing time to recanalization, improving thrombectomy techniques, and refining imaging-based patient selection. However, these approaches do not directly target the underlying mechanisms of IRI, which involve multiple interconnected biological pathways.

Emerging therapeutic strategies aim to protect the endothelium and preserve microvascular integrity, particularly through stabilization of the endothelial glycocalyx. Nanomedicine-based approaches offer potential for targeted delivery of ROS-responsive scavengers and real-time imaging of redox dynamics in ischemic tissue [77,78,79,80,81,82]. Restoration of nitric oxide signaling represents another promising strategy; NO, via activation of soluble guanylyl cyclase (sGC) and cyclic GMP production, regulates vasodilation, supports endothelial integrity, and modulates inflammatory responses [83,84,85,86,87,88,89,90,91,92,93,94,95,96,97,98,99].

Importantly, microvascular injury in AIS is sustained by a feed-forward loop involving oxidative stress, enzymatic endothelial glycocalyx degradation, and endothelial activation, leading to persistent BBB disruption, edema formation, and neuroinflammation. Even after technically successful recanalization, endothelial dysfunction and microvascular obstruction may limit effective tissue reperfusion and neurological recovery. Pre-existing comorbidities further increase susceptibility to reperfusion-related endothelial injury. Chronic hypertension promotes vascular remodeling and oxidative stress, while obesity-associated adipokines maintain a pro-inflammatory endothelial state. These conditions enhance vascular susceptibility to injury, thereby lowering the threshold for endothelial damage and increasing the risk of adverse outcomes [10,11,12,13,14,15,16,17,18,19,20,21,22,23,24,25,26,27,28,29,30,31,32,33,34,35,36,37,38,39,40,41,42,43,44,45,46,47,48,49,50,51,52,53,54,55,56,57,58,59,60,61,62,63,64,65,66,67,68,69,70,71,72,73,74,75,76,77,78,79,80,81,82,83,84,85,86,87,88,89,90,91,92,93,94,95,96,97,98,99,100,101,102,103]. Taken together, these mechanisms highlight the central role of endothelial and endothelial glycocalyx integrity in determining outcomes after AIS. Therapeutic strategies aimed at reducing oxidative stress, preserving the endothelial glycocalyx, and restoring NO signaling represent key directions for future research and potential adjuncts to reperfusion therapy [83,84,85,86,87,88,89,90,91,92].

### 2.6. Systemic and Inflammatory Cascades in Acute Ischemic Stroke 

Among the several neuropathological processes that define ischemic stroke, a strong and ongoing inflammatory response plays a crucial role in the development of brain damage. Acute disruption of the BBB is linked to inflammation after ischemia, which results in adverse neurological manifestations [104,105,106,107,108,109]. The shedding of the endothelial glycocalyx leads to enhanced leukocyte adhesion, elevated vascular permeability thus raising local neuroinflammation as well as systemic inflammatory results in SIRS after AIS [28,29,30,31,104,105,106,107,108,109]. In addition to causing localized inflammation in the brain, ischemic stroke also quickly triggers aforementioned SIRS, which is typified by the activation of peripheral inflammatory mediators and immune cells. Brain tissue experiences hypoxia because of perfusion failure, which sets off an inflammatory reaction due to the imbalance of ionic equilibrium ultimately leading to neuronal excitotoxicity, oxidative stress and lipid peroxidation, eventually leading to irreversible neurological deterioration and neuronal damage [104,105,106,107,108,109].

Glial cell-mediated disruption brought on by neuroinflammation following brain ischemia results in further excitotoxicity and the generation of ROS from the immune cells and brain. By establishing the neuroinflammatory tone, microglia and astrocytes, the brain’s immune cells, have a dual function in the CNS traumas. Induced by hypoxia, the necrotic brain cells are triggered to release DAMPs, to initiate microglia phenotypic switching to their M1 state (pro-inflammatory). Pro-inflammatory mediators such as tumor necrosis factor-alpha (TNF-α), chemokines (CXCL1, CCL5, CCL2), interleukin-6 (IL-6), IL-1β, MMP, and ROS are all amplified in the M1 state. After AIS, microglia contribute to the anti-inflammatory response by transforming from M1 to M2 phenotype, which aids in neuronal healing and repair [18,19,20,21,22,23]. Following endothelial glycocalyx degradation, BBB disruption aids the penetration of circulating inflammatory mediators into the ischemic parenchyma. Therefore, coordinating microglial activation and neuroinflammatory signaling [14,15,16,17,24,25,50,51,52,53,54,55].

Complementing inflammatory mediators, there is a release of peripheral inflammatory markers such as neutrophils, lymphocytes, and monocytes. Within 6 to 12 h after a stroke, neutrophils quickly penetrate ischemic brain tissue, reaching their peak 2–7 days later and remaining there for at least 2 weeks. By releasing inflammatory mediators (neutrophil extracellular traps) and MMPs, they intensify damage by rupturing the BBB and fostering neurotoxicity [104,105,106,107,108,109]. These mechanisms are promoted by prior endothelial glycocalyx loss [50,51,52,53,54,55,104,105,106,107,108,109]. Neutrophils have two distinct phenotypes: one being pro-inflammatory N1 cells which release toxic chemicals increasing NET formation, the other being protective N2 cells increasing macrophage elimination therefore reducing infarct volume. Lymphocytes are essential to the progression of stroke. Within 24 h of IS, T lymphocytes go to areas of ischemic brain. CD4^+^ and CD8^+^ subsets compromise the integrity of the BBB and harm the neurons via emitting of cytotoxic proteins and cytokines. However, through the inhibition of neurotoxic astrocyte proliferation mediated by amphiregulin, regulatory T cells are associated with an attenuated infarct volume and greater efficacy. Initially after a stroke, monocytes travel to IS brain areas, and classical monocytes produce pro-inflammatory cytokines, tumor necrosis factor and MMP-2/9, which interfere with BBB tight junctions and worsen the injury through the P2X4R and CCL2/CCR2 pathways. To oppose inflammation and encourage healing, non-classical monocytes exude IL-10 [104,105,106,107,108,109].

### 2.7. Clinical Relevance of Endothelial Dysfunction and Endothelial Glycocalyx Integrity in Stroke Outcomes

BBB dysfunction is a hallmark of many neurological disorders, including AIS, and plays a central role in secondary brain injury. Following ischemia and reperfusion, endothelial dysfunction represents an early manifestation of vascular injury, characterized by activation of inflammatory pathways and impairment of barrier integrity. Astrocytes and brain microvascular endothelial cells rapidly produce pro-inflammatory cytokines and chemokines, including TNF-α and interleukins, which upregulate endothelial adhesion molecules. This promotes leukocyte adhesion and transmigration, as well as degradation of extracellular matrix components and tight junction proteins, ultimately leading to BBB disruption and amplification of neuroinflammation [24,25].

Therapeutic strategies aimed at preserving BBB integrity focus on stabilizing tight junction proteins and reducing inflammatory signaling, thereby limiting leukocyte infiltration and endothelial activation [24,25]. Clinically, endothelial dysfunction can be assessed using circulating biomarkers, including adhesion molecules such as selectins, cadherins, and integrins, which reflect endothelial activation and leukocyte–endothelium interactions. In addition, non-invasive vascular imaging techniques may provide indirect assessment of endothelial function. Pharmacological approaches, including vasodilators and renin–angiotensin system modulators, as well as emerging therapies such as endothelial progenitor cells, have been explored for their potential to restore endothelial integrity and promote vascular repair. Importantly, endothelial dysfunction has been associated with adverse outcomes following endovascular treatment, including an increased risk of parenchymal hematoma, highlighting its clinical relevance as both a prognostic marker and therapeutic target [93,94,95,96,97,98].

Within this context, the endothelial glycocalyx has emerged as a critical regulator of microvascular function and a key determinant of stroke outcomes. By maintaining BBB integrity, modulating vascular tone, and limiting leukocyte and platelet adhesion, the glycocalyx plays a central role in preserving neurovascular homeostasis. However, its degradation during IRI contributes to microcirculatory dysfunction, increased vascular permeability, edema formation, and propagation of inflammatory cascades. At the molecular level, oxidative stress and enzymatic activity—particularly involving heparanase and matrix metalloproteinases—drive the breakdown of essential endothelial glycocalyx components such as heparan sulfate and chondroitin sulfate. Concurrent endothelial dysfunction, including eNOS uncoupling and reduced nitric oxide bioavailability, further destabilizes the BBB and exacerbates vascular injury [83,84,85,86,87,88,89,90,91,92].

In addition, endothelial glycocalyx shedding is associated with the release of circulating biomarkers such as syndecan-1 and endothelial microparticles expressing adhesion molecules (CD31, CD144, CD62E), which reflect endothelial injury and a pro-inflammatory, pro-thrombotic state. Clinically, reduced Ssyndecan-1 levels following intravenous thrombolysis prior to mechanical thrombectomy have been associated with decreased endothelial damage, suggesting a potential role for these biomarkers in monitoring therapeutic response.

Emerging therapeutic strategies targeting endothelial glycocalyx preservation include modulation of enzymatic degradation pathways, antioxidant approaches such as N-acetylcysteine, and interventions aimed at restoring glycocalyx components and synthesis. For example, regulation of hyaluronan metabolism through modulation of hyaluronan synthase and hyaluronidase activity may help maintain glycocalyx integrity and reduce edema formation. Overall, these findings support the concept that endothelial glycocalyx integrity is a key determinant of microvascular function and clinical outcomes in AIS. Beyond recanalization alone, incorporation of endothelial-protective strategies and glycocalyx-targeted therapies may represent a promising approach to improving neurological recovery and long-term vascular outcomes following mechanical thrombectomy [83,84,85,86,87,88,89,90,91,92].

## 3. Future Insights

Stroke remains a leading cause of mortality and long-term disability worldwide, and its global burden is expected to increase further due to population aging and demographic growth. Continued efforts are therefore needed to develop safer and more effective therapies, as well as to improve early recognition and pre hospital management of ischemic stroke [100,101,102,103,104,105].

Intravenous thrombolysis with alteplase remains a standard pharmacological treatment for acute ischemic stroke, while tenecteplase is increasingly being adopted in clinical practice due to its favorable pharmacological profile and potential safety advantages. In patients with large vessel occlusion, thrombolysis may be used alone or combined with mechanical thrombectomy within established treatment windows, with extension in selected patients based on advanced imaging criteria. Imaging guided, tissue based approaches that identify salvageable brain tissue and assess collateral circulation have expanded treatment eligibility, particularly in patients with unknown or extended onset times. In parallel, research is ongoing to improve antithrombotic strategies by developing more targeted approaches that reduce bleeding risk while maintaining efficacy [110,111,112,113,114,115].

Supportive care remains essential in stroke management and plays a key role in preventing secondary injury. This includes careful blood pressure control, regulation of blood glucose levels, prevention of deep vein thrombosis, and maintenance of normal body temperature. Early mobilization is also associated with improved outcomes. Despite improvements in treatment systems and reduced delays in care, patient outcomes remain variable, indicating the need for additional therapeutic strategies [89,90,91,92,93,99,100,101,102,103].

Although extensive research has been conducted, effective neuroprotective therapies have not yet been successfully implemented in routine clinical practice. Neuroprotection aims to interrupt the cascade of ischemic injury and promote tissue preservation and recovery. Several agents that have shown promising results in preclinical studies, including sovateltide, NA-1, edaravone, 3K3-A activated protein C, human urinary kallidinogenase, and minocycline, are currently under clinical investigation, but none have yet become standard treatment. Stem cell based therapies are also being explored for their potential to provide both early neuroprotection and long-term neuroregeneration. Approaches using neural progenitor cells, mesenchymal stem cells, and bone marrow derived stem cells have shown encouraging results in experimental studies. In addition, extracellular vesicles derived from stem cells represent a promising area of research, although their clinical application is still limited.

Future research will likely focus on combining reperfusion therapies with neuroprotective and vascular targeted strategies. Such approaches may improve treatment effectiveness, reduce reperfusion related injury, and potentially extend the treatment window in selected patients, ultimately leading to better functional recovery and long-term outcomes in AIS [89,90,91,92,93,99,100,101,102,103].

Importantly, integration of endothelial glycocalyx assessment AIS management may enhance treatment selection and risk stratification. Given that glycocalyx degradation contributes to BBB instability and increases the risk of hemorrhagic transformation following reperfusion, circulating biomarkers could be incorporated into pre-reperfusion evaluation to identify patients with increased endothelial vulnerability. In such cases, biomarker-guided strategies may influence the choice and intensity of reperfusion therapy, optimization of blood pressure targets, and the early initiation of adjunctive endothelial-protective interventions. By integrating advanced neuroimaging with biomarkers of vascular integrity and targeted pharmacological approaches, AIS management may evolve from a predominantly time-based paradigm toward a precision-based, neurovascular unit–guided strategy [14,15,16,17,28,29,30,31].

Several circulating biomarkers have shown potential for predicting post-thrombectomy reperfusion injury, particularly those reflecting endothelial damage, glycocalyx degradation, inflammation, and oxidative stress. Among these, SDC-1 and heparan sulfate (HS) have emerged as key indicators of glycocalyx shedding and microvascular injury. Elevated levels of MMP-9 are consistently associated with blood–brain barrier disruption and an increased risk of hemorrhagic transformation, while inflammatory markers such as interleukin-6 (IL-6) and C-reactive protein (CRP) reflect the magnitude of the post-reperfusion inflammatory response. Markers of endothelial activation, including soluble ICAM-1 and VCAM-1, further provide insight into vascular dysfunction.

Beyond current time- and imaging-based selection algorithms, these biomarkers are increasingly being investigated in clinical studies as tools for risk stratification and outcome prediction following thrombectomy. Emerging multimodal approaches combining glycocalyx-related biomarkers with advanced imaging techniques, such as perfusion imaging and assessment of BBB permeability, may enable more precise identification of patients at risk of reperfusion injury and support individualized therapeutic strategies. However, despite their promising clinical relevance, these biomarkers are not yet part of routine clinical practice, and further validation is required to establish their predictive value and facilitate their integration into clinical decision-making [115]. Paradigm toward a precision based, neurovascular unit guided strategy [14,15,16,17,28,29,30,31].

## 4. Conclusions

AIS is a complex cerebrovascular condition in which vascular occlusion, endothelial dysfunction, and inflammation interact to determine neurological outcomes. Increasing evidence highlights the endothelial glycocalyx as a key regulator of vascular homeostasis in this condition. During ischemia, dynamic degradation of the endothelial glycocalyx promotes leukocyte adhesion, activation of coagulation pathways, disruption of the blood–brain barrier, and amplification of inflammatory cascades, thereby contributing to secondary brain injury beyond the initial ischemic insult.

Mechanical thrombectomy has fundamentally transformed the management of acute ischemic stroke caused by large vessel occlusion by enabling rapid restoration of cerebral perfusion and significantly improving functional outcomes. However, reperfusion, while essential for salvaging ischemic tissue, may also induce reperfusion-related injury characterized by oxidative stress, endothelial damage, and intensified inflammatory responses. The integrity of the endothelial glycocalyx plays a central role in modulating these processes, influencing barrier stability, immune cell infiltration, and the risk of complications such as hemorrhagic transformation and systemic inflammatory responses. The demonstrated efficacy of thrombectomy within extended time windows, supported by advanced imaging techniques, further underscores its pivotal role in modern stroke care.

In conclusion, mechanical thrombectomy remains the most effective therapeutic option for patients with AIS. While emerging approaches such as stem cell therapy, neuroprotective strategies, and nanomedicine show promise, they have not yet achieved sufficient clinical maturity. A deeper understanding of endothelial glycocalyx disruption and its interaction with inflammatory pathways may improve prognostic assessment and support the development of adjunctive therapies aimed at preserving vascular integrity and enhancing long-term neurological recovery.

## Data Availability

No new data was created or analyzed in this study. Data sharing is not applicable to this article.
